# Nurses' assessment of subsyndromal delirium and barriers to assessment: A cross‐sectional survey in the intensive care unit

**DOI:** 10.1111/jonm.13887

**Published:** 2022-11-17

**Authors:** Yan Gao, Chuanlai Zhang, Chunlian Liao, Xiuni Gan

**Affiliations:** ^1^ Nursing Department The Second Affiliated Hospital of Chongqing Medical University Chongqing China; ^2^ Department of Intensive Care Medicine The Second Affiliated Hospital of Chongqing Medical University Chongqing China; ^3^ Department of Neurology The Second Affiliated Hospital of Chongqing Medical University Chongqing China

**Keywords:** barriers, intensive care units, nurses, subsyndromal delirium

## Abstract

**Aims:**

The aims of the study are to investigate the current status of nurses' assessment of subsyndromal delirium (SSD) in the intensive care unit (ICU) and explore possible barriers to assessment.

**Background:**

SSD is a dynamic, recognizable disorder commonly seen in the ICU that can lead to poor patient outcomes. Timely recognition and management can prevent its progression.

**Methods:**

A cross‐sectional survey design was used to collect data from ICU registered nurses in southwest China. The online survey containing an analysis of the current status of SSD assessment and barriers was completed by 237 nurses.

**Results:**

A total of 51.5% of nurses chose to assess SSD using an assessment tool, the most commonly used being the Confusion Assessment Method for the Intensive Care Unit; the frequency of assessment was mostly once a day (66, 41.0%) and often at shift change (178, 87.3%). There were statistically significant differences in the barrier factor scores by assessment frequency, assessment method, status of training in SSD, ability of SSD‐related knowledge to meet clinical needs and willingness to receive SSD training.

**Conclusion:**

Our study confirms that the current state of assessment of SSD in the ICU is unsatisfactory, with nurses' lack of assessment knowledge and skills, poor organization and management, and the complexity of patients' conditions being barriers.

**Implications for nursing management:**

Nursing managers should systematically conduct training programmes on effective SSD assessment knowledge and skills, incorporate SSD assessment into the daily workflow, have standardized assessment tools, develop standardized processes and assign dedicated staff to monitor, audit and provide feedback on SSD assessments.

## 1 INTRODUCTION

Delirium is a neurocognitive disorder characterized by an acute change in cognition, attention and consciousness that results in what experts describe as brain failure (Setters & Solberg, 2017). It is very common for patients in the intensive care unit (ICU) to experience delirium, especially for patients who are receiving mechanical ventilation, for whom the incidence of delirium is as high as 80% (Shehabi et al., 2010). The onset of delirium prolongs ICU stays, increases mortality, and leaves some patients with short‐ or long‐term cognitive impairment after discharge (Devlin et al., 2018). Delirium is currently a key public health concern and a hot topic of discussion in the field of critical care medicine. Both the Diagnostic and Statistical Manual of Mental Disorders, Fifth Edition (DSM‐V) (American Psychiatric Association, 2013) and the International Classification of Diseases 11th Revision (ICD‐11) (WHO, 2018) diagnostic criteria for delirium require multiple cognitive symptoms to be present. However, it has been found in clinical practice that many patients experience one or more of the symptoms of delirium but do not meet the diagnostic criteria for delirium, which scholars have labelled subsyndromal delirium (SSD) (Levkoff et al., 1996).

Partial syndromes of delirium are not a novel concept. In the 16th century, a physician wrote that the predelirious phase (paraphrenitis) could either precede a full‐blown delirium or not, depending on the patient's constitution, the illness underlying the delirium, and the way it is treated Lipowski described a “predelirious” phase, in which patients with one or more symptoms of delirium never develop the full DSM‐defined syndrome (Lipowski, 1990). The DSM‐IV also states that individuals may manifest some but not all symptoms of delirium, which are referred to as subsyndromal presentations of delirium (American Psychiatric Association, 1994). International diagnostic authorities have taken note of this phenomenon, with the DSM‐IV describing it as a subsyndromal manifestation and coding it under the classification of “undefined cognitive impairment”; in the DSM‐V, SSD is listed under the classification of neurocognitive disorders with the term “attenuated delirium syndrome”. The term “subsyndromal delirium” appears in the ICD‐11 but is coded under a different classification than delirium, with delirium coded in chap. 6 on “mental, behavioural or neurodevelopmental disorders” and SSD coded in chap. 21. The “unclassified signs, symptoms or clinical findings” are listed under the category of “confusion”. However, neither has a clear definition of SSD.

Current scholars believe that SSD and delirium belong to the same disease spectrum and are two different states of one disease (Martínez Velilla et al., 2012; Ouimet et al., 2007). Studies have shown that SSD and delirium share similar influencing factors (Cole et al., 2013; Levkoff et al., 1996). Similar to delirium, SSD also contributes to adverse outcomes such as prolonged length of hospital stay, institutionalization, decreased quality of life and cognitive impairment. The severity ranges between delirium and no delirium (Cole et al., 2013; Gao et al., 2022).

It is important to note that the progression and regression of delirium may be intermediate to SSD (Cole et al., 2003) and that there are no effective pharmacological treatment options for SSD (Devlin et al., 2018). SSD is a dynamically fluctuating disease that can progress to delirium or regress. Therefore, the early and accurate identification of SSD is important, as it can facilitate early and effective prevention and treatment, thereby reducing the adverse effects of SSD. However, in a cross‐sectional survey involving 1521 patients in the ICU in 47 countries, it was found that only 42% of health care workers used an SSD detection tool correctly (Morandi et al., 2017). Nurses were only 15% to 31% sensitive to the key features of SSD, with an incorrect identification rate of 81% (Inouye et al., 2001). There is a lack of research on the current status of SSD assessment of ICU nurses and a lack of research on identifying and analysing the barriers to SSD assessment. Therefore, this study aims to understand the current status of SSD assessment among ICU nurses and analyse the barriers to SSD assessment to provide reasonable suggestions to promote the practice of SSD assessment in ICUs.

## 2 METHODS

### 2.1 Design

A cross‐sectional survey design was used.

### 2.2 Aims

This study aimed to (1) determine the current status of nurses' assessment of SSD in the ICU, (2) explore the barriers to SSD assessment and (3) provide sound recommendations to facilitate the practice of SSD assessment in the ICU. For these purposes, the following research questions were examined regarding nurses in China:

Q1. What is the current status of the assessment of SSD in the ICU?
Q2. What are the barriers that influence the assessment of SSD in the ICU?
Q3. What are their personal, professional and assessment status‐related characteristics that make a significant difference in terms of factors that are barriers to SSD assessment?
Q4. What do nursing practice managers, as well as practitioners, need to be aware of to address these barriers and improve practice in the assessment of SSD in the ICU?

**FIGURE 1:**
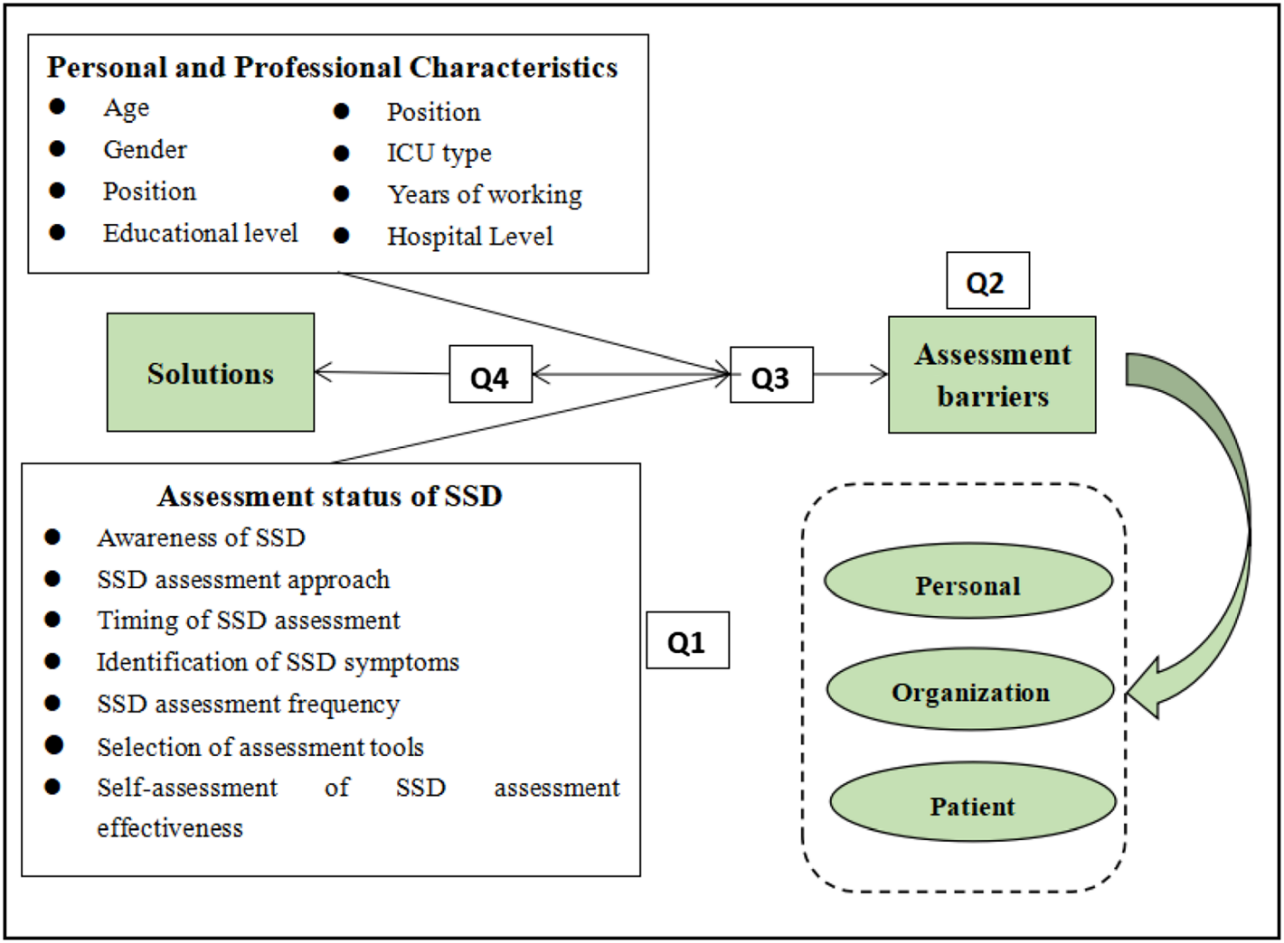
Research model. Abbreviations: ICU, intensive care unit; Q, research question; SSD, subsyndromal delirium

In line with the research questions, the research model structured using the related concepts and variables is shown in Figure 1.

### 2.3 Sample and participants

The study population was drawn from seven hospitals in southwest China. Inclusion criteria were as follows: (1) registered nurses currently working in the ICU; (2) consent to participate in this study. Exclusion criteria included trainee and intern nurses. The sample was recruited through a convenience sampling method, and a total of 237 nurses participated in the survey. The sample size was calculated using PASS 2021 software (Version 21.0, NCSS Crop., USA), based on the formulae of the two independent samples *t*‐test (Chow et al., 2018) and one‐way ANOVA (Borm et al., 2007), with the mean of each group in the pretest as a parameter (*α* = .05, *β* = .1). The power analysis showed the required sample size of nurses was a maximum of 111 cases, and considering the 20% invalid questionnaire and sampling error, it was reasonable to distribute 237 cases.

### 2.4 Data collection

The researcher's unit is a tertiary care public hospital qualified to hold specialist nurse training in ICUs. Each year, the intensive care medicine unit recruits specialist nurse trainees from outside the unit for a 3‐month training period to obtain a qualification as specialist nurses in intensive care medicine. The survey was conducted from 9 February to 28 April 2022 using Questionnaire Star (an online crowdsourcing platform in China). The questionnaire was distributed in two steps: first, the questionnaire was distributed to the WeChat groups (a widely used social media platform in China) of 40 specialist trainees, inviting them to participate in the survey; second, the research team used convenience sampling to contact the managers of seven specialist trainees' organizations, and the managers distributed the questionnaire to their WeChat groups.

#### 2.4.1 Study instruments

The survey consisted of four parts: (i) guiding words, (ii) demographic and professional information, (iii) an ICU nurse SSD assessment status questionnaire and (iv) a SSD assessment barrier questionnaire. The questions used in the survey were based in part on previous research (Li et al., 2016). Prior to the start of this study, five experts in the fields of neurocritical care, critical care medicine and nursing were invited to review the content of the questionnaire. Among the five experts, three held master's degrees and two held bachelor's degrees; the experts included two supervising nurse practitioners, one deputy chief nurse practitioner, one chief nurse practitioner, and one deputy chief physician. The survey was reviewed twice by the experts to refine the questions and responses and to incorporate suggested changes into the survey. An online version of the survey was later sent to 20 practice nurses whose responses and feedback were used to assess formatting, logic checks, clarity of responses and the time taken to answer questions.

##### Guiding words

The guiding words explained to the respondent the content and purpose of the study and asked the respondent to provide informed consent. Guidance on the dos and do nots of completing the questionnaire was provided, and it was explained that participation was completely anonymous and voluntary and would not involve the disclosure of personal information.

##### Demographic and professional information

The sociodemographics of participants were collected. This information included the following: participants' age, gender, educational level, position, years of working in the ICU, position, hospital level and type of ICU.

##### SSD assessment status questionnaire

This questionnaire consisted of 12 questions, including the following: (1) Have you ever learned about SSD? (2) Have you ever cared for a subject with SSD? (3) What are the symptoms that determine SSD? (4) When and how often do you assess SSD at work? (5) What is the timing and frequency of SSD assessment? (6) Are you capable of SSD assessment? (7) Does SSD‐related knowledge meet clinical needs? (8) Are you willing to participate in SSD training? and (9) What is your preferred training approach?

##### SSD assessment barriers questionnaire

The section was informed by cognitive, behavioural, social influence and marketing theories (Grol & Grimshaw, 2003) and contained 28 items in three dimensions: the individual, organizational and patient levels. At the individual level, there were 14 items on nurses' awareness, attitudes, knowledge and competence in SSD assessment; at the organizational level, there were 9 items on human resource arrangements, feedback and evaluation systems, relevant institutional requirements, and procedures for SSD assessment; and at the patient level, there were 5 items on the assessment of sedated and mechanically ventilated patients and patients' conditions and lack of cooperation. Each item was rated on a 5‐point Likert scale, where *strongly disagree*, *disagree*, *unclear*, *agree* and *strongly agree* were scored as 1, 2, 3, 4, and 5 points, respectively; the higher the score was, the more relevant the implementation process was to the factor. The content validity indexes were determined to be 0.94 for the individual level, 0.93 for the organizational level, 0.93 for the patient level and 0.94 for the total questionnaire. The results of the presurvey of 20 nurses showed Cronbach's alpha coefficients of .869 at the individual level, .911 at the organizational level, .868 at the patient level and .941 for the total questionnaire.

### 2.5 Data analysis

Data were exported in an Excel file (Microsoft Corp., Redmond, WA, USA) and analysed by SPSS 26.0 statistical software (IBM Corp., Group NY). The significance level was considered to be *p* ≤ .05. Descriptive analysis was applied to the general data. Count data are expressed as frequencies and percentages; measurement data are expressed as the means ± standard deviations. The mean ± standard deviation is used to express the score of each dimensional barrier factor. Differences between groups were compared for dichotomous variables using two independent samples *t*‐tests for group comparisons and one‐way ANOVA for multiclass variables.

### 2.6 Ethical considerations

Ethical approval was obtained from the Ethics Committee of the Second Hospital of Chongqing Medical University (Date: 26 August 2021, Number: 2021‐74). Nurses who agreed to participate in the study were contacted via social media and provided their data. Participants' consent was obtained on the first page of the online data collection tool, which contained information regarding the research aim, scope, and ethical issues. After completing the questionnaire, each participant received payment; there was no hierarchical relationship between the participants and the researchers.

## 3 RESULTS

### 3.1 Participants

A total of 237 ICU nurses participated in this study. The nurses were mainly from secondary and tertiary public hospitals, with Grade A tertiary hospitals accounting for the majority (207, 87.3%), and most of the nurses were from general ICUs (168, 70.9%). The majority of the nurses were female (214, 90.3%). The age range was mostly between 26–30 (103, 43.5%) and 31–40 years (87, 36.7%). With regard to the level of education, the majority of the respondents had a bachelor's degree (199, 84.0%), followed by an associate degree (30, 12.7%) and a master's degree (8, 3.4%). In addition, the majority of the survey respondents had a junior title (119, 50.2%) and worked in clinical nursing (200, 84.4%) (Table 1).

**TABLE 1:** Demographic and professional information of the participants (*N* = 237)

Characteristics	Option	Answer (*n*)	Frequency (%)
Gender	Male	23	9.7
	Female	214	90.3
Age (years)	18–25	41	17.3
	26–30	103	43.5
	31–40	87	36.7
	41–50	6	2.5
Education	Diploma/associate's degree	30	12.7
	Bachelor's degree	199	84.0
	Master's degree	8	3.4
Years of clinical nursing	<0.5	30	12.7
	0.5–1	42	17.7
	2–5	55	23.2
	6–10	67	28.3
	11–20	42	17.7
	>20	1	0.4
Primary position	Staff nurse	200	84.4
	Educating nurses	29	12.2
	Head nurse	8	3.4
Professional title	Nurse	53	22.4
	Nurse practitioner	119	50.2
	Nurse‐in‐charge	59	24.9
	Professor of nursing	6	2.5
Work department	GICU	168	70.9
	SICU	17	7.2
	MICU	37	15.6
	EICU	9	3.8
	NICU	4	1.7
	RICU	2	0.8
Hospital level	Grade A tertiary hospital	207	87.3
	Grade B tertiary hospital	5	2.1
	Grade A secondary hospital	22	9.3
	Grade B secondary hospital	3	1.3

Abbreviations: EICU, emergency intensive care unit; GICU, general intensive care unit; MICU, medicine intensive care unit; *n*, number; NICU, neurological intensive care unit; RICU, respiratory intensive care unit; SICU, surgical intensive care unit.

### 3.2 Nurses' assessment of SSD in ICUs

The survey found that 70% of nurses had cared for subjects with SSD in the course of their work, indicating that SSD is relatively common in the ICU. The timing of the assessment was most frequent at shift handover (178, 87.3%) or when the patient's cognition changed (166, 81.4%), with a frequency of once a day being the most common (66, 41.0%). In terms of assessment methods, 51.5% of the nurses chose to use an assessment tool, with CAM‐ICU being the most commonly used assessment tool; a further 35.8% chose to draw on their own clinical experience for assessment. However, 37.1% and 41.4% of nurses were unsure of whether the assessment tool and clinical experience would be accurate in assessing SSD, respectively (Table 2).

**TABLE 2:** Current status of subsyndromal delirium assessment in ICU nurses

Questions	Options	Answers (*n*)	Frequency (%)
Have you ever known SSD (*n* = 237)	Yes	138	58.2
	No	61	25.7
	Unsure	38	16.0
Have cared for a patient with SSD (*n* = 237)	Yes	166	70.0
	No	33	13.9
	Unsure	38	16.0
SSD evaluation frequency at work (*n* = 161)	No assessment	23	14.3
	Once a day	66	41.0
	Twice a day	32	19.9
	Three times a day	19	11.8
	More	21	13.0
Timing of SSD assessment at work (*n* = 204)	Routine assessment	108	52.9
	Shift changeover	178	87.3
	Cognitive changes	166	81.4
	Mood swings	82	40.2
	Use of cognitive impairment drugs	115	56.4
	Others	4	2.0
SSD assessment methods (*n* = 204)	Personal clinical experience	73	35.8
	Using the assessment tools	105	51.5
	Through doctors' consultations	26	12.7
Whether clinical experience can accurately assess SSD (*n* = 73)	Yes	23	31.5
	No	20	27.4
	Unsure	30	41.1
Assessment tools used (*n* = 105)	CAM‐ICU	61	58.1
	ICDSD	10	9.5
	RASS	34	32.4
Whether the assessment tool can accurately assess SSD (*n* = 105)	Yes	59	56.2
	No	7	6.7
	Unsure	39	37.1
Whether trained in SSD assessment (*n* = 237)	Yes	63	26.6
	No	153	64.6
	Unsure	21	8.9
Date of last training (*n* = 63)	Less than 1 month	13	20.6
	Less than 3 month	10	15.9
	Less than 6 month	15	23.8
	More than 1 year	25	39.7
Does your SSD‐related knowledge meet clinical care needs (*n* = 237)	Yes	71	30.0
	No	111	46.8
	Unsure	55	23.2
Willingness to be trained in SSD knowledge (*n* = 237)	Yes	211	89.0
	No	26	11.0
Preferred training method (*n* = 237)	Studying at school	1	0.4
	Academic conferences, lectures	60	25.3
	SSD related learning course	91	38.4
	Self‐learning	10	4.2
	Specialist nurse training	31	13.1
	Communication between colleagues	4	1.7
	Online education platform for medical/nursing professions	40	16.9

Abbreviations: CAM‐ICU, the Confusion Assessment Method for the Intensive Care Unit; ICU, intensive care unit; ICDSD, the Intensive Care Delirium Screening Checklist; *n*, number; RASS, Richmond Agitation–Sedation Scale; SSD, subsyndromal delirium.

**FIGURE 2:**
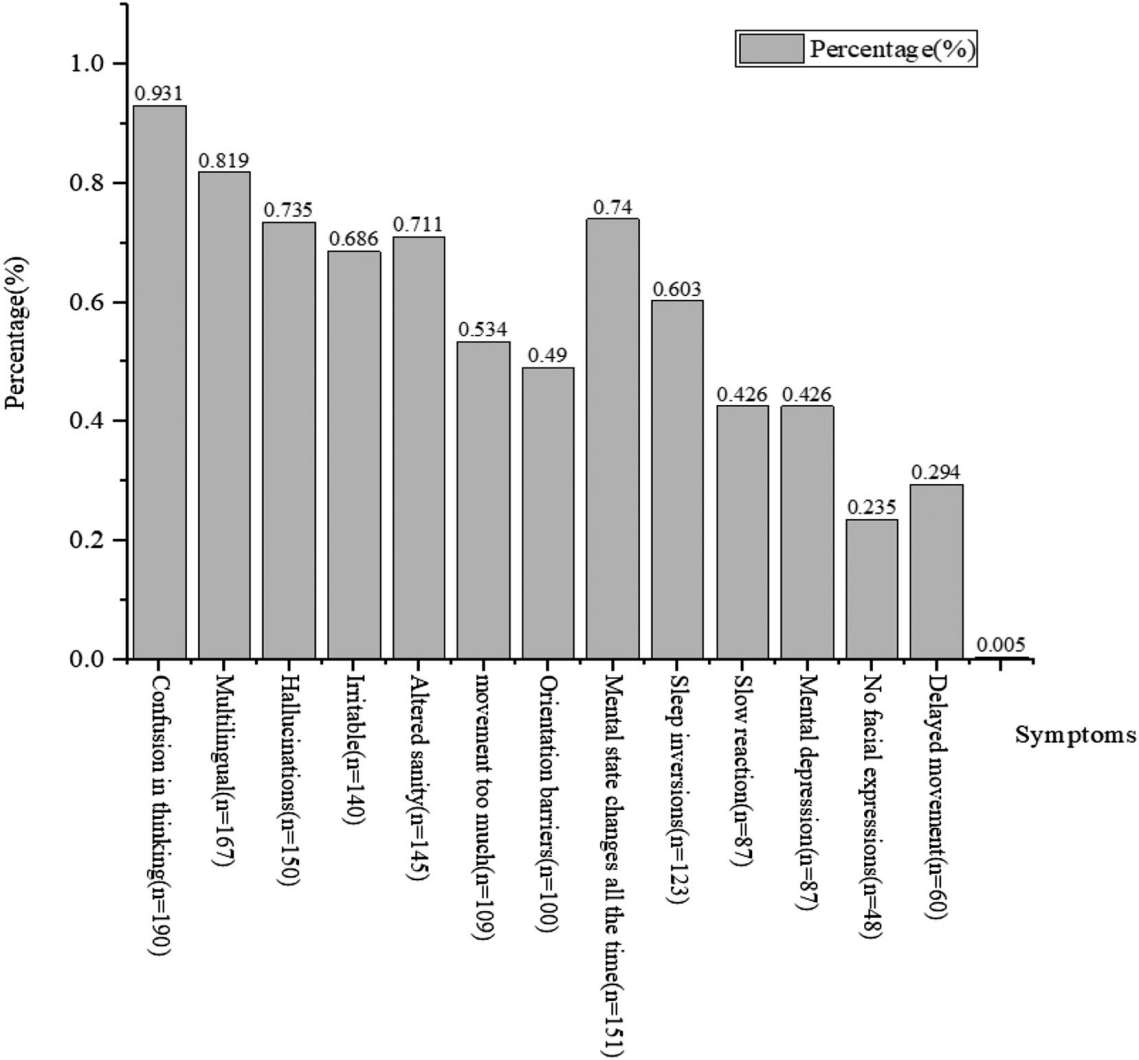
Determine the clinical signs of a subject experiencing subsyndromal delirium

When the ICU nurse identified SSD, the five most common clinical signs that indicated the presence of SSD were confused thinking, multilingualism, rapid changes in mental status, hallucinations and loss of sanity, while the five least common clinical signs were expressionlessness, delayed movement, depression, slow reaction and disorientation, as shown in Figure 2.

In addition, regarding whether the nursing competencies of the ICU met the clinical care needs of patients with SSD, only 30.0% of nurses felt that their competencies met the requirements. Only 26.6% of the nurses had received training in SSD‐related knowledge. The majority of the nurses wanted to attend SSD training (211, 89.0%), and the most preferred method of training was to attend an SSD course (91, 38.4%).

### 3.3 Barriers to assessing of SSD in ICUs

The means and standard deviations of the dimensions of each barrier factor are shown in Table 3. The results showed that the differences by age, gender, education level, years of ICU experience, title, position, ICU type and hospital level were not statistically significant in the SSD assessment between individual‐level barrier factors and total barrier factor scores. However, the scores for the organizational level barrier factors showed statistically significant differences by education level (*p* = .012) and level of health care facility (*p* = .028). In addition, the gender differences (*p* = .028) and years of working (*p* = .05) for the ICU nurses in the patient‐level barrier factor scores showed statistical significance (Appendix S1).

**TABLE 3:** Subsyndromal delirium assessment barrier factor score

Item	Ranking	Barriers	Score (mean ± *SD*)
Individual level	1	Lack of knowledge in the differential diagnosis of SSD	3.61 ± 0.898
	2	Not fully grasping the care of SSD prevention	3.51 ± 0.909
	3	In the ICU, it is difficult to achieve timely and repeated assessments of SSD	3.50 ± 0.900
	4	Assessment tools for SSD not fully mastered	3.44 ± 0.966
	5	It is believed that current assessment tools are not good enough to diagnose SSD	3.42 ± 0.911
	6	No access to an authoritative knowledge of SSD	3.32 ± 0.942
	7	Lack of understanding of the specific clinical presentation of SSD	3.18 ± 0.998
	8	Not trusting the results of your assessment of SSD	3.14 ± 0.937
	9	People who are not aware of the high risk of SSD occurring	3.13 ± 1.042
	10	Not knowing the main risk factors for SSD	3.10 ± 1.018
	11	Increased workload	2.82 ± 1.015
	12	SSD assessment is not considered to improve patient prognosis	2.64 ± 1.018
	13	SSD assessment is a matter for the doctor, not yourself	2.24 ± 0.925
	14	SSD assessment is not important to focus on	2.17 ± 1.012
	Total average score at the individual level		3.09 ± 0.674
Organization level	1	Lack of time for SSD assessment due to heavy workload in the department	3.40 ± 1.014
	2	Lack of multidisciplinary teamwork	3.32 ± 1.013
	3	The department does not provide process specifications for SSD assessments	3.30 ± 1.086
	4	Lack of incentives	3.29 ± 1.027
	5	No SSD management rules in the department	3.28 ± 1.033
	6	SSD assessment skills training is not provided in the department	3.25 ± 1.066
	7	The section does not require routine assessment for SSD	3.21 ± 1.076
	8	The section does not provide the relevant tools for SSD assessment	3.16 ± 1.106
	9	The results of the SSD assessment are not taken seriously by the department and are not fed back on time	2.92 ± 1.073
	Total average score at the organizational level		3.24 ± 0.877
Patient level	1	Difficulties in implementing SSD assessment for intubated patients	3.77 ± 0.769
	2	Difficulties in implementing SSD assessment for sedated patients	3.77 ± 0.808
	3	Difficulty distinguishing SSD from mental cognitive disorders such as dementia	3.65 ± 0.884
	4	Subjective lack of patient cooperation makes assessment difficult	3.62 ± 0.812
	5	Difficulties in assessing patients with severe neurological impairment	3.78 ± 0.765
	Total mean score at the patient level		3.72 ± 0.686

Abbreviations: ICU, intensive care unit; *SD*, standard deviation; SSD, subsyndromal delirium.

Differential comparison of the current status of assessment of SSD in the ICU revealed statistically significant differences in barrier factor scores by frequency of assessment, mode of assessment, training status in SSD‐related knowledge, self‐assessed ability of SSD‐related knowledge to meet clinical needs and willingness to receive SSD training (Table 4). In contrast, there was no significant difference between the scores on the barrier factors for the different assessment tools and the time of the most recent training.

**TABLE 4:** Univariate analysis of factors impairing the assessment of subsyndromal delirium in ICU nurses

	*n*	Score (mean ± *SD*)	Statistical values	*p*
Assessment frequency			10.598 ^a^	<.001^**^
No assessment	23	3.69 ± 0.522		
Once a day	66	3.28 ± 0.522		
Twice a day	32	2.85 ± 0.628		
Three times a day	19	3.08 ± 0.567		
More	21	2.75 ± 0.724		
Assessment methods			15.708 ^a^	<.001^**^
Personal clinical experience	73	3.47 ± 0.514		
Using assessment tools	105	3.01 ± 0.623		
By doctor's consultation	26	3.40 ± 0.484		
Assessment tools used			1.346 ^a^	.265
CAM‐ICU	61	3.08 ± 0.572		
ICDSD	10	2.80 ± 0.593		
RASS	34	2.92 ± 0.707		
Whether trained in SSD			18.754 ^a^	<.001^**^
Yes	63	2.86 ± 0.748		
No	153	3.40 ± 0.502		
Unsure	21	3.30 ± 0.633		
Date of last SSD training			1.583 ^a^	.203
Less than 1 month	13	2.94 ± 0.763		
Less than 3 month	10	2.45 ± 1.076		
Less than 6 month	15	2.79 ± 0.557		
More than 1 year	25	3.03 ± 0.656		
Does SSD‐related knowledge meet clinical needs			15.872 ^a^	<.001^**^
Yes	71	2.99 ± 0.776		
No	111	3.47 ± 0.506		
Unsure	55	3.13 ± 0.481		
Willingness to be trained in SSD knowledge			−4.943^b^	<.001^**^
Yes	211	3.18 ± 0.604		
No	26	3.80 ± 0.587		

Abbreviations: CAM‐ICU, the Confusion Assessment Method for the Intensive Care Unit; ICDSD, the Intensive Care Delirium Screening Checklist; *n*, number; RASS, Richmond Agitation–Sedation Scale; *SD*, standard deviation; SSD, subsyndromal delirium.

^a^ *t*‐test coefficient.

^b^ Analysis of variance (ANOVA); *p*, statistical significance.

* *p* ≤ .05.

** *p* ≤ .001.

## 4 DISCUSSION

This study surveyed 237 registered nurses in the ICUs of seven hospitals and found that the current status of SSD assessment was unsatisfactory and that there were many factors that prevented ICU nurses from assessing SSD.

The survey showed that 70% of the nurses had cared for patients with SSD in the course of their work, indicating that SSD is common in the ICU. This is similar to the findings of previous studies, where the incidence of SSD in the ICU ranged from 31.4% to 49.7% (Azuma et al., 2019; Bastos et al., 2019; Yamada et al., 2018). However, it is worth noting that only 58.2% of the nurses in the study had knowledge of SSD, which is similar to the Miao FF study (Miao, 2020), indicating a low level of awareness of SSD among ICU nurses.

Regarding the choice of SSD assessment method, 51.5% of the nurses chose to use an assessment tool, with the most commonly used assessment tool being the CAM‐ICU (61, 58%). This is in line with the guideline opinion recommendations (Devlin et al., 2018). The CAM‐ICU was originally validated in 96 adult patients at Vanderbilt University Medical Center (USA) in a medical or coronary ICU. Compared with the reference standard used for diagnosing delirium, the CAM‐ICU had a sensitivity of 100% and 93%, specificity of 98% and 100% and high interrater reliability (*κ* = 0.96; 95% confidence interval CI [0.92, 0.99]) (Ely et al., 2001). The CAM‐ICU is simple and easy to use and provides a rapid assessment of delirium and SSD, but the assessment is qualitative dichotomous and does not show the dynamics of the patient's severity in terms of scores. Clinical practice guidelines also recommend that the Intensive Care Delirium Screening Checklist (ICDSC) identify patients with SSD (Devlin et al., 2018; Stollings et al., 2021). The ICDSC was initially validated in 93 patients at Maisonneuve‐Rosemont Hospital in Montreal, Quebec, Canada. The predictive sensitivity of ICDSC was 99%, but the specificity was only 64% (Bergeron et al., 2001). The changes in assessment values can also clearly show the changes in the patient's condition. Therefore, the combined use of the CAM‐ICU and ICDSC can help to solve the problem of the dichotomous assessment of the CAM‐ICU scale without visual changes in values and improve the problem of the low specificity of the ICDSC scale by combining qualitative and quantitative aspects in the assessment process, but its clinical effectiveness still needs to be further tested. In addition, 37.1% of nurses who used the assessment tool were still unsure about the effectiveness of the tool in assessing SSD, indicating that the importance, use and proficiency of the SSD assessment tool among health care staff could be strengthened and improved.

Notably, our survey found that 35.8% of nurses used clinical experience to identify SSD, but 41.1% of these were not sure if it was accurately assessed, which to some extent reflects a low level of SSD awareness, in line with previous studies (Miao, 2020; Ning et al., 2019; Wang et al., 2020). In our study, only 26.6% of nurses had received training related to SSD assessment, and 70% felt that their competencies were not adequate for clinical needs. This reminds managers of the importance of training in SSD‐related knowledge and improving the ability of ICU nurses to recognize and manage SSD symptoms.

ICU nurses assessing SSD currently have many barriers. The individual‐level barriers included nurses' perceptions, attitudes, knowledge and competencies regarding SSD assessment. The results showed that the top three barrier factors were lack of knowledge related to the differential diagnosis of SSD, incomplete knowledge of nursing measures for SSD prevention and difficulty in achieving timely and repeated assessment of SSD in the ICU. Distinguishing SSD from other psychiatric cognitive disorders remains difficult as there is still a lack of uniform criteria for defining SSD and its diagnosis still requires the use of delirium assessment tools (Meagher & Trzepacz, 2007). Guidelines recommend the use of nonpharmacological means to treat SSD, but specific nonpharmacological interventions for treatment still need further clinical validation (Devlin et al., 2018). More research is needed to explore SSD assessment skills, as there is a lack of assessment and status surveys on SSD assessment skills. Furthermore, in the survey on the importance of SSD assessment, nurses did not consider SSD to be unimportant and they believed that SSD assessment would have a favourable impact on patient prognosis, which is consistent with the findings of several studies (Miao, 2020; Ning et al., 2019; Wang et al., 2020). This indicates that nurses have a positive attitude towards SSD assessment, which provides strong support for the development of follow‐up training.

The top three organizational‐level barriers were a busy department and lack of time to conduct SSD assessments, a lack of multidisciplinary teamwork and a department that did not provide process specifications for SSD assessments. Chang and Yu (2016) showed that the work of ICU nursing staff conformed to a heavier workload and that nursing work was significantly associated with quality of care, prompting nursing managers to rationalize nursing staffing in the ICU and reduce the workload of nursing staff. Departments do not give enough attention to SSD assessment, and the standardization process, management rules and multidisciplinary teamwork need to be further improved. Interestingly, there were statistically significant differences in the organizational level barrier factors by nurses' level of education and the level of the hospital in which they were worked (*p* < .05). As the level of education increased, so did the scores for the organizational level barrier factor. This may be because nurses with higher levels of education are more likely to be in managerial positions and are more likely to view problems from an organizational management perspective. In addition, the higher the hospital level was, the lower the score on the organizational barrier factor. This may be because higher‐ranking hospitals have more well‐established departments.

Patient‐level scores were high for all barrier factors. Patients undergoing intubation and sedation can be influenced by the assessor's subjective factors, as their consciousness is more difficult to assess (Robinson et al., 2013). This can serve as a reminder for health care professionals to be diligent in observing these patients so that they are not missed. In addition, there was a significant difference in the assessment of SSD at the patient level between nurses of different genders (*p* = .028), with the majority of nurses in this survey being female, but the reasons for this need to be further investigated and explained.

### 4.1 Limitations

There are several limitations to this study. First, the survey was conducted in only one city in China, so there are limitations to the generalizability of the results. Second, the results of the study covered only six common types of adult ICUs, as well as data not received, for example, cardiac ICUs and paediatric ICUs. Third, although the sample of hospital nurses was appropriate, only a small number of nurses from tertiary and secondary B hospitals participated. Fourth, this study used a quantitative research approach to prospectively identify some of the barriers, and although the questionnaire had fill‐in‐the‐blank questions for the study participants to fill in other barriers, the number filled in was not very high. Therefore, it is recommended that future qualitative studies be conducted to explore other barriers to SSD assessment from multiple perspectives, including those of nursing staff, clinicians and psychiatrists.

## 5 CONCLUSION

This study investigated and analysed the current situation of and barriers to the assessment of SSD among ICU nurses in the hospital, suggesting that the current situation of SSD assessment needs to be improved, with lack of knowledge and competence among nurses, high work pressure among ICU nurses, lack of attention from nursing managers and the complexity of patients' conditions being the main barriers to assessment. An effective SSD assessment knowledge and skills training programme should be systematically implemented, and the workflow should be improved at the organizational level to provide conditions and support for SSD assessment, facilitate the practice of SSD assessment in the ICU, identify SSD at an early stage and intervene at an early stage to reduce the risk of SSD advancing to delirium for ICU patients.

## 6 IMPLICATIONS FOR NURSING MANAGEMENT

Nursing managers should emphasize SSD assessment practices and systematically conduct effective training programmes on SSD assessment knowledge and skills in the ICU, including an overview of the background knowledge of SSD assessment, the use of assessment tools and precautions and reinforcement of assessment difficulties and key points, especially for sedated and intubated patients; training methods should include regular SSD‐related training, supplemented by academic conferences and lectures; in addition, posters or pamphlets on SSD assessment can be displayed or distributed to enhance the learning atmosphere. Departments should integrate SSD assessment into their routine workflow, have standardized assessment tools, develop a standardized process, define the frequency of assessment and recording methods and arrange for a dedicated person to be responsible for monitoring, auditing and providing feedback on SSD assessment in order to standardize and promote the practice of SSD assessment from an organizational level. In addition, the SSD assessment process should be integrated into the intensive care information system with reminders and alerts, or intelligent SSD assessment tools, such as an app, should be developed for bedside use by nurses to improve their compliance and efficiency in SSD assessment.

There is currently no clear definition of SSD and no specific assessment tool, and its diagnosis and assessment are based on the core symptoms of delirium or assessment tools. Pharmacological treatment has not been shown to be effective in alleviating SSD, and nonpharmacological interventions have been recommended by national and international scholars, although the effectiveness of nonpharmacological interventions needs to be further tested. Therefore, exploratory and empirical studies could be conducted in the future to improve the understanding of SSD and to establish uniform and targeted diagnostic criteria for SSD. In addition, effective interventions and nonpharmacological treatment options for SSD, such as cluster strategies, early rehabilitation, daily arousal combined with music therapy, cognitive training and other nonpharmacological treatments for delirium, could be explored in the future.

## CONFLICT OF INTEREST

None. This research did not receive any specific grant from funding agencies in the public, commercial or not‐for‐profit sector.

## ETHICS STATEMENT

Ethical approval was obtained from the Ethics Committee of the Second Hospital of Chongqing Medical University (Date: 26 August 2021, Number: 2021‐74).

## Supporting information


**Appendix S1** Univariate analysis of demographic information as a factor in the assessment of barriers to subsyndromal delirium (Mean±SD)

## Data Availability

The datasets used or analysed during the current study are available from the corresponding author on reasonable request.
