# Supplementary material for: Nurses' assessment of subsyndromal delirium and barriers to assessment: A cross‐sectional survey in the intensive care unit
Source: J Nurs Manag. 2022 Nov 17;30(8):4491–502. doi: 10.1111/jonm.13887 (PMC10099329; doi:10.1111/jonm.13887)
Supplement: Supplementary file 1 — Appendix S1 Univariate analysis of demographic information as a factor in the assessment of barriers to subsyndromal delirium (Mean±SD) [file JONM-30-4491-s001.docx]

**Appendix 1 Univariate analysis of demographic information as a factor in the assessment of barriers to subsyndromal delirium (Mean±SD )**

**·Gender differences**

| **Barriers** | **Male** | **Female** | **t** | **P** |
| --- | --- | --- | --- | --- |
| Individual-level | 2.95$\boldsymbol{\pm}$0.859 | 3.10$\boldsymbol{\pm}$0.652 | -1.006 | .316 |
| Organization-level | 3.06$\boldsymbol{\pm}$1.048 | 3.26$\boldsymbol{\pm}$0.857 | -1.010 | .314 |
| Patient-level | 4.02$\boldsymbol{\pm}$0.736 | 3.69$\boldsymbol{\pm}$0.674 | 2.215 | .028* |
| Overall | 3.18$\boldsymbol{\pm}$0.802 | 3.26$\boldsymbol{\pm}$0.612 | -.561 | .576 |

**·Age differences**

| **Barriers** | **18～25** | **26～30** | **31～40** | **41～50** | **F** | **P** |
| --- | --- | --- | --- | --- | --- | --- |
| Individual-level | 3.27$\boldsymbol{\pm}$0.087 | 3.03$\boldsymbol{\pm}$0.070 | 3.07$\boldsymbol{\pm}$0.073 | 3.06$\boldsymbol{\pm}$0.168 | 1.312 | .271 |
| Organization-level | 3.37$\boldsymbol{\pm}$0.744 | 3.19$\boldsymbol{\pm}$0.948 | 3.25$\boldsymbol{\pm}$0.872 | 2.94$\boldsymbol{\pm}$0.390 | .603 | .613 |
| Patient-level | 3.85$\boldsymbol{\pm}$0.548 | 3.73$\boldsymbol{\pm}$0.744 | 3.66$\boldsymbol{\pm}$0.680 | 3.60$\boldsymbol{\pm}$0.551 | .841 | .473 |
| Overall | 3.41$\boldsymbol{\pm}$0.496 | 3.21$\boldsymbol{\pm}$0.692 | 3.23$\boldsymbol{\pm}$0.624 | 3.12$\boldsymbol{\pm}$0.340 | 1.106 | .347 |

**·Difference in years of experience**

| **Barriers** | **<0.5** | **0.5~1** | **2~5** | **6~10** | **11~20** | **>20** | **F** | **P** |
| --- | --- | --- | --- | --- | --- | --- | --- | --- |
| Individual  -level | 3.27$\boldsymbol{\pm}$0.497 | 3.20$\boldsymbol{\pm}$0.747 | 2.97$\boldsymbol{\pm}$0.620 | 3.03$\boldsymbol{\pm}$0.771 | 3.09$\boldsymbol{\pm}$0.595 | 3.43 | 1.173 | .323 |
| Organization-level | 3.44$\boldsymbol{\pm}$0.637 | 3.38$\boldsymbol{\pm}$0.959 | 3.03$\boldsymbol{\pm}$0.914 | 3.24$\boldsymbol{\pm}$0.883 | 3.21$\boldsymbol{\pm}$0.868 | 3.44 | 1.171 | .324 |
| Patient  -level | 3.69$\boldsymbol{\pm}$0.612 | 4.00$\boldsymbol{\pm}$0.703 | 3.55$\boldsymbol{\pm}$0.667 | 3.55$\boldsymbol{\pm}$0.667 | 3.67$\boldsymbol{\pm}$0.587 | 4.00 | 2.257 | .050* |
| Overall | 3.40$\boldsymbol{\pm}$0.412 | 3.40$\boldsymbol{\pm}$0.713 | 3.09$\boldsymbol{\pm}$0.613 | 3.22$\boldsymbol{\pm}$0.703 | 3.23$\boldsymbol{\pm}$0.54 | 3.54 | 1.606 | .159 |

**·Education differences**

| **Barriers** | **Diploma/Associate’s degree** | **Bachelor’s degree** | **Master’s degree** | **F** | **P** |
| --- | --- | --- | --- | --- | --- |
| Individual-level | 3.05$\boldsymbol{\pm}$0.763 | 3.08$\boldsymbol{\pm}$0.669 | 3.29$\boldsymbol{\pm}$0.417 | .413 | .662 |
| Organization-level | 3.12$\boldsymbol{\pm}$0.965 | 3.22$\boldsymbol{\pm}$0.861 | 4.13$\boldsymbol{\pm}$0.354 | 4.545 | .012* |
| Patient-level | 3.58$\boldsymbol{\pm}$0.843 | 3.72$\boldsymbol{\pm}$0.664 | 4.15$\boldsymbol{\pm}$0.351 | 2.223 | .111 |
| Overall | 3.17$\boldsymbol{\pm}$0.711 | 3.24$\boldsymbol{\pm}$0.623 | 3.71$\boldsymbol{\pm}$0.284 | 2.456 | .088 |

**·Difference in professional title**

| **Barriers** | **Nurse** | **Nurse Practitioner** | **Nurse-in**  **-charge** | **Professor of Nursing** | **F** | **P** |
| --- | --- | --- | --- | --- | --- | --- |
| Individual-level | 3.10$\boldsymbol{\pm}$0.606 | 3.12$\boldsymbol{\pm}$0.744 | 3.03$\boldsymbol{\pm}$0.615 | 2.87$\boldsymbol{\pm}$0.219 | .466 | .707 |
| Organization-level | 3.17$\boldsymbol{\pm}$0.840 | 3.25$\boldsymbol{\pm}$0.945 | 3.29$\boldsymbol{\pm}$0.811 | 3.15$\boldsymbol{\pm}$0.335 | .207 | .891 |
| Patient-level | 3.76$\boldsymbol{\pm}$0.671 | 3.72$\boldsymbol{\pm}$0.763 | 3.73$\boldsymbol{\pm}$0.535 | 3.33$\boldsymbol{\pm}$0.484 | .694 | .557 |
| Overall | 3.24$\boldsymbol{\pm}$0.545 | 3.27$\boldsymbol{\pm}$0.725 | 3.24$\boldsymbol{\pm}$0.528 | 3.04$\boldsymbol{\pm}$0.133 | .267 | .849 |

**·Position differences**

| **Barriers** | **Staff nurse** | **Educating nurse** | **Head nurse** | **F** | **P** |
| --- | --- | --- | --- | --- | --- |
| Individual-level | 3.09$\boldsymbol{\pm}$0.690 | 3.11$\boldsymbol{\pm}$0.618 | 3.04$\boldsymbol{\pm}$0.509 | .027 | .973 |
| Organization-level | 3.21$\boldsymbol{\pm}$0.883 | 3.43$\boldsymbol{\pm}$0.910 | 3.28$\boldsymbol{\pm}$0.497 | .775 | .462 |
| Patient-level | 3.72$\boldsymbol{\pm}$0.705 | 3.82$\boldsymbol{\pm}$0.559 | 3.35$\boldsymbol{\pm}$0.510 | 1.484 | .229 |
| Overall | 3.24$\boldsymbol{\pm}$0.645 | 3.34$\boldsymbol{\pm}$0.585 | 3.17$\boldsymbol{\pm}$0.437 | .356 | .701 |

**·Difference in hospital level**

| **Barriers** | **Grade A tertiary hospital** | **Grade B tertiary hospital** | **Grade A secondary hospital** | **Grade B secondary hospital** | **F** | **P** |
| --- | --- | --- | --- | --- | --- | --- |
| Individual-level | 3.06$\boldsymbol{\pm}$0.679 | 3.14$\boldsymbol{\pm}$0.484 | 3.28$\boldsymbol{\pm}$0.665 | 3.60$\boldsymbol{\pm}$0.352 | 1.304 | .274 |
| Organization-level | 3.17$\boldsymbol{\pm}$0.893 | 3.58$\boldsymbol{\pm}$0.547 | 3.68$\boldsymbol{\pm}$0.635 | 3.89$\boldsymbol{\pm}$0.509 | 3.087 | .028* |
| Patient-level | 3.72$\boldsymbol{\pm}$0.702 | 3.52$\boldsymbol{\pm}$0.502 | 3.75$\boldsymbol{\pm}$0.593 | 3.67$\boldsymbol{\pm}$0.577 | .157 | .925 |
| Overall | 3.21$\boldsymbol{\pm}$0.648 | 3.35$\boldsymbol{\pm}$0.325 | 3.49$\boldsymbol{\pm}$0.475 | 3.70$\boldsymbol{\pm}$0.372 | 1.861 | .137 |

**·Difference in work department**

| **Barriers** | **GICU** | **SICU** | **MICU** | **EICU** | **NICU** | **RICU** | **F** | **P** |
| --- | --- | --- | --- | --- | --- | --- | --- | --- |
| Individual-  level | 3.04$\boldsymbol{\pm}$0.693 | 3.28$\boldsymbol{\pm}$0.698 | 3.19$\boldsymbol{\pm}$0.569 | 3.16$\boldsymbol{\pm}$0.369 | 2.86$\boldsymbol{\pm}$1.241 | 3.43 | .826 | .532 |
| Organization-  level | 3.19$\boldsymbol{\pm}$0.901 | 3.18$\boldsymbol{\pm}$0.732 | 3.36$\boldsymbol{\pm}$0.853 | 3.62$\boldsymbol{\pm}$0.431 | 2.97$\boldsymbol{\pm}$1.395 | 4.00 | .955 | .446 |
| Patient-level | 3.77$\boldsymbol{\pm}$0.648 | 3.72$\boldsymbol{\pm}$0.459 | 3.59$\boldsymbol{\pm}$0.819 | 3.78$\boldsymbol{\pm}$0.612 | 3.00$\boldsymbol{\pm}$1.414 | 3.00 | 1.817 | .110 |
| Overall | 3.22$\boldsymbol{\pm}$0.648 | 3.33$\boldsymbol{\pm}$0.567 | 3.32$\boldsymbol{\pm}$0.575 | 3.42$\boldsymbol{\pm}$0.276 | 2.92$\boldsymbol{\pm}$1.298 | 3.54 | .628 | .678 |

Abbreviations:SD, standard deviation; t, t test coefficient; F, Analysis of variance (ANOVA); GICU, General Intensive Care Unit; SICU, Surgical Intensive Care Unit; MICU, Medicine Intensive Care Unit; EICU, Emergency Intensive Care Unit; NICU, Neurological Intensive Care Unit; RICU, Respiratory Intensive Care Unit; *p ≤ 0.05.
